# Defining a Blush

**Published:** 1892-11

**Authors:** 


### Defining a Blush.

A Cincinnati physician defines a blush as follows: "A blush is a temporary erythema and calorific effulgence of the physiognomy, ætiologized by the perceptiveness of the sensorium when in a predicament of unequilibrity from a sense of shame, anger or other cause, eventuating in a paresis of the vasomotor nervous filaments of the facial capillaries, whereby, being divested of their elasticity, they are suffused with radiance, emanating from an intimidated præcordia." That settles it. She's from Boston.

### Character.

A man passes for what he is worth. What he is, engraves itself on his face, on his form, on his fortunes, in letters of light which all men may read but himself. Concealment avails him nothing, boasting nothing. There is confession in the glances of our eyes, in our smiles, in salutations, and the grasp of hands.—Emerson.

